# Twin Town in South Brazil: A Nazi's Experiment or a Genetic Founder Effect?

**DOI:** 10.1371/journal.pone.0020328

**Published:** 2011-06-08

**Authors:** Alice Tagliani-Ribeiro, Mariana Oliveira, Adriana K. Sassi, Maira R. Rodrigues, Marcelo Zagonel-Oliveira, Gary Steinman, Ursula Matte, Nelson J. R. Fagundes, Lavinia Schuler-Faccini

**Affiliations:** 1 Departamento de Genética, Instituto de Biociências, Universidade Federal do Rio Grande do Sul, Porto Alegre, Rio Grande do Sul, Brazil; 2 National Institute of Science and Technology in Populational Medical Genetics (INAGEMP), Porto Alegre, Brazil; 3 Universidade Federal do Pampa, São Gabriel, Rio Grande do Sul, Brazil; 4 Departamento de Biologia Geral, Instituto de Biociências, Universidade Federal de Minas Gerais, Belo Horizonte, Minas Gerais, Brazil; 5 Hospital de Clínicas de Porto Alegre, Porto Alegre, Rio Grande do Sul, Brazil; 6 Department of Biochemistry, Touro College of Osteopathic Medicine, New York, New York, United States of America; University of Utah, United States of America

## Abstract

Cândido Godói (CG) is a small municipality in South Brazil with approximately 6,000 inhabitants. It is known as the “Twins' Town” due to its high rate of twin births. Recently it was claimed that such high frequency of twinning would be connected to experiments performed by the German Nazi doctor Joseph Mengele. It is known, however, that this town was founded by a small number of families and therefore a genetic founder effect may represent an alternatively explanation for the high twinning prevalence in CG. In this study, we tested specific predictions of the “Nazi's experiment” and of the “founder effect” hypotheses. We surveyed a total of 6,262 baptism records from 1959–2008 in CG catholic churches, and identified 91 twin pairs and one triplet. Contrary to the “Nazi's experiment hypothesis”, there is no spurt in twinning between the years (1964–1968) when Mengele allegedly was in CG (*P* = 0.482). Moreover, there is no temporal trend for a declining rate of twinning since the 1960s (*P* = 0.351), and no difference in twinning among CG districts considering two different periods: 1927–1958 and 1959–2008 (*P* = 0.638). On the other hand, the “founder effect hypothesis” is supported by an isonymy analysis that shows that women who gave birth to twins have a higher inbreeding coefficient when compared to women who never had twins (0.0148, 0.0081, respectively, *P* = 0.019). In summary, our results show no evidence for the “Nazi's experiment hypothesis” and strongly suggest that the “founder effect hypothesis” is a much more likely alternative for explaining the high prevalence of twinning in CG. If this hypothesis is correct, then this community represents a valuable population where genetic factors linked to twinning may be identified.

## Introduction

The etiology of twin births in humans is still largely unclear and is the subject of a number of investigations [1]–[4]. Traditionally, twins are classified as monozygotic (MZ) and dizygotic (DZ). MZ twins are developed when an embryo splits soon after fertilization. DZ twins occur when two separate oocytes, released during the same menstrual period, are fertilized by separate sperm cells [1]. MZ is rarer than DZ twinning except in Japan [5] and no maternal, genetic, or environmental factors have been associated to it so far [6]. On the other hand, DZ twinning is a phenomenon of complex etiology, in which both genetic predisposition and environmental factors play a role [7]. Although familial aggregation of DZ twins has been known for a long time, few genes have been associated with DZ twinning, including the receptor of FSR hormone (*FSHr*), growth differentiation factor 9 (*GDF9*), methylenetetrahydrofolate reductase (*MTHFR*), etc. [8]–[10]. Non-genetic factors such as advanced maternal age, increased parity, lactation, diet, higher maternal height body mass index and race are observed also as risk factors for DZ twinning too [11].

Twinning rates shows a wide geographical and temporal variation, being extremely rare in Asian populations (5–6 in 1,000 maternities) and more frequent in Sub-Saharian populations (23 in 1,000 maternities) [11]. Previous studies show that twinning rates in European countries started to decline around 1900, but have increased steadily from the 1970s onwards [12], possibly as a result of both increased maternal age and a more widespread use of assisted reproductive technology (ART) procedures [11], [13], [14]. Even in a single continent such as Europe variations on twinning rates between different countries are observed [15]. The variability on twinning rates has being assigned to the variation in DZ twinning rates, as MZ twinning has a constant prevalence around the world and time (4 in 1,000 maternities) [16].

Cândido Godói (CG) is a small town in South Brazil (27°57′07″S; 54°45′07″W) with approximately 6,000 inhabitants. It is known as the “Twins' Town” due to its high rate of twin births. According to the Brazilian Ministry of Health, between 1994 and 2006, around 2% of the live births in CG were twins, compared to an average of 1% for the whole country [17]. However, twinning may not be equally distributed throughout the municipality. In 1994, the twinning birth rate in Linha São Pedro (LSP), a small district of CG was estimated as 10% [18]. CG and LSP were both founded at the beginning of the Twentieth Century by a few families of German ancestry coming from other German-founded towns in Rio Grande do Sul. Presently, the population of LSP is less than 600 inhabitants and most are catholic [19]. The reasons for the higher twinning rate in CG in general, and in LSP in particular, however, are still unclear.

Recently, a controversial theory was raised by an Argentinean journalist [20] who wrote a book alleging a possible link between the twinning phenomenon in CG and supposed experiments of the Nazi physician, Joseph Mengele. According to Camarasa, Mengele could have lived and worked as a physician in CG in the beginning of the 1960s, after living in Buenos Aires. Even though Camarasa's suppositions were not based on any actual historical records available [21], his story has caught attention of the international press, which created a worldwide “fuzz” around the “Brazilian Twin Town” in many countries such as the United Kingdon (http://www.dailymail.co.uk/news/worldnews/article-1126504/The-Twins-Brazil-Did-Nazi-doctor-Mengele-Angel-Death-cause-twin-surge-South-American-town.html), Brazil (http://revistaepoca.globo.com/Revista/Epoca/0,EMI24803-15228,00-NAZISTAJOSEFMENGELECRIOUCIDADEDOSGEMEOSNORIOGRANDEDOSULDIZLIVR.html) and USA (http://news.nationalgeographic.com/news/2009/11/091125-nazi-twins-brazil-mengele.html).

In this study, we surveyed baptism records in CG and LSP to evaluate temporal trends to test the predictions of the “Nazi's experiment hypothesis”. More specifically, we asked the following questions: 1. Is there any temporal and geographical variation in twinning in CG (and LSP)? 2. Is there any increase of the twinning rate around the 60 s or late 60 s, when Joseph Mengele was supposedly working as a physician there? We also searched for alternative explanations for the higher twinning rate in CG. Given the known history of CG, we suspected that a genetic founder effect may be involved in the increased twinning rate in CG (and LSP). To test this hypothesis, we asked, using isonymy methods, whether twin's mothers have a higher inbreeding coefficient as compared to women who never gave birth to twins. Our results clearly show that contrary to the “Nazi's experiment hypothesis” there is no peak on the twinning rate around the 60 s. On the other hand, the isonymy analysis supports the hypothesis that a founder effect is a much more likely explanation for the higher prevalence of twinning in LSP and in CG as a whole.

## Materials and Methods

### Ethics Statement

This study used only secondary public data for analysis, which was treated anonymously. This research project was approved by the Hospital de Clinicas de Porto Alegre Ethics Committee under the protocol number 09-359. Written informed consent was obtained for all participants before interviews were conducted.

### Twinning patterns within CG

Live births were surveyed from baptism records available in CG Catholic churches. These records include the child first name, family names of both parents, sex, and the locality (district) where the family lives. The earliest available baptism records date from 1927, but complete and reliable data were available only from 1959 onwards. Since there is a suggestion that LSP may have an especially high twinning frequency [18], we used a chi-square test to assess whether LSP has a higher twinning rate as compared to the remaining CG districts (CG-LSP) considering 1959 to 2008. Because maternal age (MA) is a known factor affecting twinning [11] we compared MA distribution in LSP and CG-LSP using data obtained from interviews to test if women living in LSP have children later in life, thus increasing the chance of twin births. Mean maternal age between these groups was compared using a Student *t*-test adjusted for unequal variances.

### Temporal tendencies in LSP and CG

According to Camarasa [20], Mengele would have arrived in CG around 1963 and visited the city until 1968, but the exact timing for his stay is uncertain. To evaluate the hypothesis that Mengele's stay in CG would have increased the twinning rate in the city, we used a chi-square test to compare the occurrence of twin births between the period of 1964–1968 and the remaining years.

To have a more detailed view on the temporal variations of twinning in CG, we used a G-test for trends to ask if there is any temporal trend over the frequency of twin births occurring after 1959, grouped by intervals of five years. We tested for temporal trends in twin births for CG as a whole, for LSP only, and for CG-LSP. The “Nazi's experiment hypothesis” predicts a peak of twinning around the late 1960s followed by either a constant or abrupt decline on the twinning rate towards the average level in the Brazilian population, since the increase in twinning obtained by Mengele would be effective only on the short-term.

Finally we used a chi-square test to evaluate if the relative frequency of twinning between LSP and CG-LSP was held constant when two periods are considered: 1927–1958 and 1959–2008. Because the earliest records (up to 1959) are only reliable for live born twins – but not for overall births only the relative twinning frequency in different CG districts could compared. All statistical tests, odds ratios, and their 95% confidence interval were computed using BioEstat 5.0 [22].

### MZ/DZ ratio

The proportion of the MZ twinning rate (*MZr*) over the DZ twinning rate (*DZr*) was estimated using Weinberg's differential method [23] in which *DZr* can be estimated by doubling the number of opposite sex twin pairs (*OS*) and dividing by the total number of maternities (*N*): *DZr = 2OS/N*, while *MZr* can be estimated by subtracting the number of *OS* twins from the number of same sex (*SS*) twins and dividing by *N: MZr* = (*SS*−*OS*)/*N*. Because we only have the total number of maternities for the period 1959–2008, this was the period considered for *MZr* and *DZr* estimation. The corrected variances for *MZr* and *DZr* (Var(*MZr*) and Var(*DZr*), respectively) was estimated as in [24]. We compared *MZr* and *DZr* between LSP and CG-LSP using a binomial test. Finally, we used the chi-square distribution to test if *MZr* and *DZr* are equally increased in LSP compared to CG-LSP using the absolute number of MZ and DZ twin pairs estimated by the numerator of Weinberg formula. As the total number of maternities is not necessary for this estimation we used the full birth records from 1927–2008 to increase statistical power.

### Pedigree and isonymy

To have a more detailed picture of the pedigree structure for families where a high twinning rate is reported compared to families without familial history of twinning, we interviewed 42 women from independent households who gave birth to twins (cases) and 101 women from independent households who only had single births (controls). All 143 women were residents from CG. The aim of this questionnaire was to collect further data that would provide a better description of these women and to access other possible risk factors for twinning. More specifically, we asked familiar data, including surnames from both parents and history of twinning in their family, and questions about their lifestyle. For each household, we used the software Progeny 7.0® to draw pedigrees. We then used the surname data to calculate isonymy, a quantity that makes use of the frequency of surnames in a given population to measure deviations from panmixia [25]. In this sense, isonymy is related to the inbreeding coefficient *F* [26], which was estimated as in [27]. Analyses of population structure by isonymy methods have been carried out in many human populations [28]–[32]. The “founder effect hypothesis” predicts that the higher twinning rate in CG reflects, at least in part, a genetic founder effect which happened when CG was settled by few families of German descent in the beginning of the 20^th^ century. Thus, if such founder effect affects the twinning rate in CG we expect a higher inbreeding coefficient for cases. We calculated *F* for cases (*F_cases_*) and controls (*F_controls_*), and the statistical significance of this difference was assessed by 1,000 permutations. The reported *P-*value thus represents the proportion of permutations resulting in a ratio *F_cases_*/*F_controls_* equal or higher than the observed.

## Results

**Figure 1:**
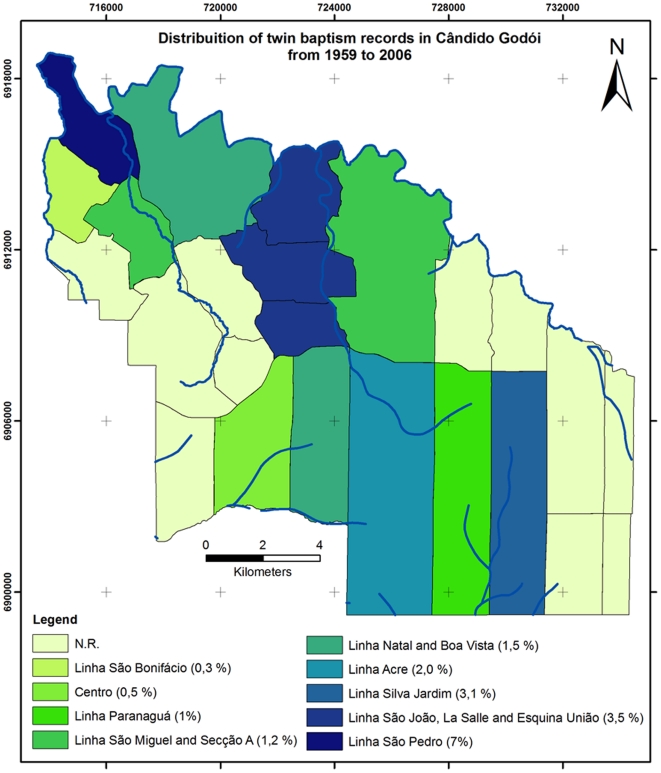
Districts of Cândido Godói with their twin births registration.

From 1959 to 2008 there were a total of 6,262 baptisms in 14 CG districts, including 91 pairs of twins and one triplet. Table 1 presents the geographical distribution of baptism records in CG according to the district where the parents lived. Using total baptism records we estimated the frequency of twinning in these districts. LSP shows a frequency of live born twins of 7.0% compared to 1.5% in CG as a whole. No other district presented frequencies above 3.5% (Table 1, Figure 1). It is noteworthy that while LSP contributes only 7.5% to all baptism records, it contributes almost 1/3 (33/92) to all twin births registered in CG, representing an odds ratio of 7.3 (CI 95% 4.75–11.38) for LSP compared to the remaining districts in CG (*P*<0.0001) (Table 2). There was no difference in maternal age between LSP and CG-LSP (*P* = 0.3036; MA_LSP_ = 26.10±4.71 years; MA_CG-LSP_ = 26.87±5.88 years (mean±SD), suggesting that differences in maternal age is unlikely to explain the higher twinning rate in LSP.

**Table 1:** Distribution of twin baptism records in Cândido Godói from 1959 to 2008.

District	Total baptisms		Twin records		Prevalence of twinning*
	N	%	N	%	%
Centro	2,548	40. 7	13	14.1	**0.5**
Linha São Miguel	512	8.2	6	6.5	**1.2**
Linha São Pedro	469	7.5	33	35.9	**7.0**
Sede Boa Vista	588	9.4	9	9.8	**1.5**
Linha São Bonifácio	398	6.4	1	1.1	**0.3**
Linha Secção A	406	6.5	5	5.4	**1.2**
Linha Acre	293	4.7	6	6.5	**2.0**
Linha Paranaguá	196	3.1	2	2.2	**1.0**
Linha Natal	338	5.4	5	5.4	**1.5**
Linha São João, La Salle and Esquina União	168	2.6	6	6.5	**3.5**
Linha Godói Centro	217	3.5	2	2.2	**0.9**
Linha Silva Jardim	129	2.1	4	4.3	**3.1**
**TOTAL**	**6,262**	**100**	**92**	**100**	**1.5**

*Calculated as twin records/total baptism records.

**Table 2:** Twin and single baptism records in Linha São Pedro (LSP) and in others Cândido Godói's (CG-LSP) districts, from 1959–2008.

	Twin births	Single births
**LSP**	33 (35.9%)	436 (7.1%)
**CG – LSP**	59 (64.1%)	5,735 (92.9%)
**CG (total)**	92 (100.0%)	6,170(100.0%)

Relative Risk for twinning in LSP = 7,3 (CI 95%: 4.75–11.38).

Chi-square test *P*<0.0001.

**Figure 2:**
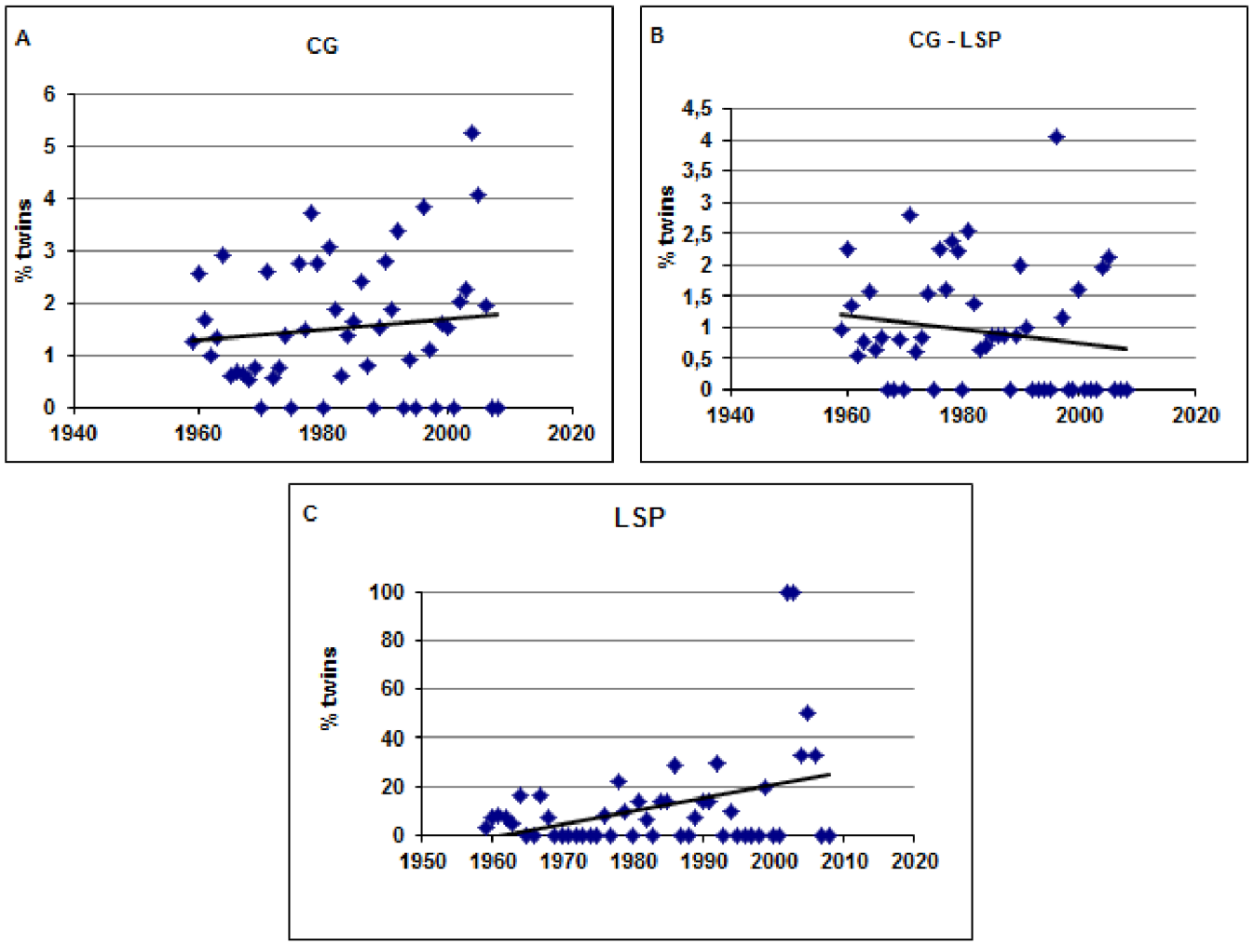
Temporal twinning tendencies. 2a: Temporal twinning tendencies in Cândido Godói; 2b: Temporal twinning tendencies in Cândido Godói, excluded Linha Sao Pedro; 2c: Temporal twinning tendencies in Linha Sao Pedro only.

There is no increase on the twinning rate in CG between the period 1964–1968 and the remaining years (Table 3, *P* = 0.482). These results are consistent when only LSP or CG-LSP are analyzed (*P* = 0.772, *P* = 0.294, respectively). There is also no temporal variation on the relative frequency of twinning between LSP and CG-LSP, considering the periods of 1927–1958 and 1959–2008 (Table 4, *P* = 0.638), suggesting that LSP has been historically the major contributor for the overall higher twinning rate in CG. The test for temporal trends in the frequency of twin births reveals an interesting pattern (Table 5). There is no evidence for any temporal trend in either CG as a whole (Figure 2A; *P* = 0.351) or CG-LSP (Figure 2B; *P* = 0.486). In contrast, for LSP alone there is a tendency for an increase on the twinning rate across time (Figure 2C; *P* = 0.001). Importantly, this trend goes in the opposite direction of that expected according to the “Nazi's experiment hypothesis”, whose predictions are also rejected in all other tests we performed.

**Table 3:** Twin baptism records between 1964–1968 and in the remainders years in Linha Sao Pedro (LSP) and in others Cândido Godói's (CG-LSP) districts.

CG	Twins	No-twins	*P value*
**1964–1968**	9	778	0.4815
**Others years**	80	5395	
**CG-LSP**	**Twins**	**No-twins**	
**1964–1968**	4	718	0.2940*
**Others years**	53	5018	
**LSP**	**Twins**	**No-twins**	
**1964–1968**	5	60	0.7724**
**Others years**	27	377	

*P*yates*.

**P*williams*.

**Table 4:** Twin baptism records before and after 1958 in Linha Sao Pedro (LSP) and in others Cândido Godói's (CG-LSP) districts.

	1927–1958	1959–2008
**LSP**	11 (31.4%)	33 (35.9%)
**CG – LSP**	24 (68.6%)	59 (64.1%)
**CG (total)**	92 (100.0%)	6,170(100.0%)

Qui-square test: *P* = 0.638.

**Table 5:** Temporal distribution of twin baptism records from 1959 to 2008, in Linha São Pedro (LSP), in other districts of Cândido Godói (CG-LSP) and Cândido Godói as a whole (CG).

Period	LSP		CG-LSP		CG	
	Twins/single births	%	Twins/single births	%	Twins/single births	%
1959–1963	4/84	4.8	12/965	1.2	16/1049	1.5
1964–1968	5/60	8.3	4/718	0.6	9/778	1.2
1969–1973	0/67	0.0	8/749	1.1	8/816	1.0
1974–1978	3/54	5.6	10/641	1.6	13/695	1.9
1979–1983	3/53	5.7	10/723	1.4	13/776	1.7
1984–1988	4/34	11.8	4/596	0.7	8/630	1.3
1989–1993	7/36	19.4	4/471	0.8	11/507	2.2
1994–1998	0/30	0	4/390	1.0	4/420	1.0
1999–2003	3/9	33.0	1/257	0.4	4/266	1.5
2004–2008	4/9	44.4	2/224	0.9	6/233	2.6

**Figure 3:**
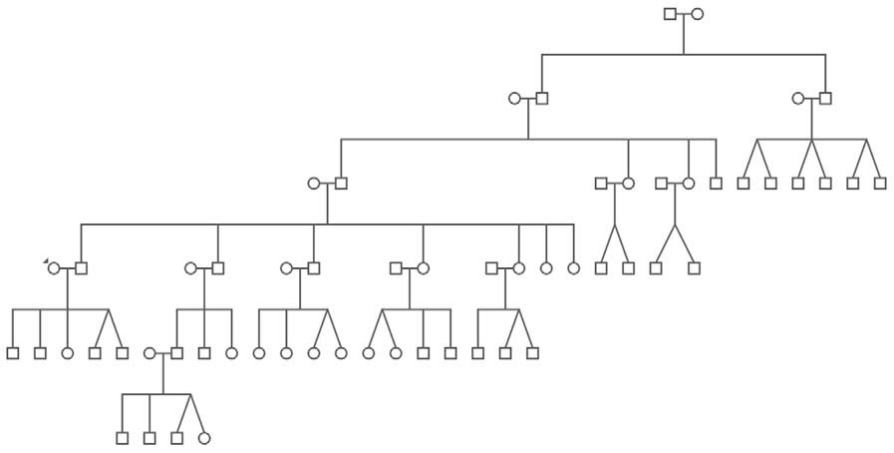
Illustrative pedigree of one family living in Linha São Pedro.

Table 6 shows for both LSP and CG-LSP the numbers of *SS* and *OS* for the period between 1927–1958, and *SS*, *OS* and total births for the period between 1959–2008. For this last period, *DZr* equals 3,84% for LSP but only 0,48% in CG-LSP, while *MZr* equals 2,98% in LSP and only 0,64% in CG-LSP. This difference is highly significant for both *DZr* and *MZr* (P<0,0001 for both comparisons). To test if *MZr* and *DZr* are increased by the same amount in LSP we used the numerator of Weinberg formula to estimate that 32 DZ twin pairs and 11 MZ twin pairs were born in LSP between 1927–2008, while during the same period 42 DZ twin pairs and 46 MZ twin pairs were born in CG-LSP. This difference is significant (*P* = 0.004). Thus, even though both *DZr* and *MZr* are increased in LSP, such increase seems to affect DZ twin births more strongly than MZ twin births. Since genetic factors have been only associated with DZ twinning, this result may be taken as a suggestion that genetic factors are responsible for the increased twinning rate seen in CG (for which LSP is the major contributor). Such notion is reinforced from the pedigrees resulting from the reported familiar history, which allows us to observe that twin birth is recurrent for several families living in CG (Figure 3). Twenty-eight out of 42 mothers of twins had female sisters, and in five out of these 28 kindreds (17.8%), recurrence of twin births was observed. The total number of sisters of index mothers was 85, five of them having twins, which means an overall recurrence chance of having twins in females by 5.9%.

**Table 6:** Estimation of DZ twinning rate (*DZr*), MZ twinning rate (*MZr*) and the absolute number of DZ and MZ twin pairs in LSP and CG-LSP.

	LSP				CG-LSP			
	1927–1958		1959–2008		1927–1958		1959–2008	
	*SS*	*OS*	*SS*	*OS*	*SS*	*OS*	*SS*	*OS*
	4	7	9	23	16	7	14	51
**Total births**	Unknown		469		Unknown		5793	
**DZr** *			3.8380%				0.4833%	
**Var(DZr)** *			0.0161%				0.0002%	
**MZr** *			2.9851%				0.6387%	
**Var(MZr)** *			0.0144%				0.0002%	

*SS*, same sex twin pairs; *OS*, opposite sex twin pairs.

*estimated using data from 1959–2008 only.

Finally, by analyzing the distribution of surnames between cases and controls, we found that the estimated *F* for cases is statistically higher than the estimated *F* for controls (*P* = 0.019; Table 7), suggesting that, as predicted by the founder effect hypothesis, women having twins in CG are more genetically related than women having single births.

**Table 7:** Inbreeding coefficient by Isonomy (F) from cases and controls.

Sample	Number of Individuals	Number of Surnames	F
**Cases**	42	85	0.0148
**Controls**	101	196	0.0081

*P* = 0.019.

## Discussion

Our results provide a robust refutation to the hypothesis that the high twinning rate in CG is due to Nazi's experiments in the 1960s, and strongly suggest that a genetic founder effect occurred during the settlement of CG is a much better alternative to explain this phenomenon. On other hand, we confirmed that the high prevalence of twins in CG is particularly concentrated in LSP and that this is a historical trend. Importantly, this increase on the twinning rate at LSP is not due to fertility treatments, because these techniques have been extremely rare in Brazil due to its high costs, especially in rural areas like CG.

One of the limitations of this study is that it is based on baptism registries and not on official birth certificates or hospital births records. However, birth certificates in Brazil provide reliable information for twinning only after 1990, and old hospital records were not available in CG. Moreover, in small communities such as CG was quite common in the first decades of the 20^th^ century for women to have babies at home or to attend to hospitals in the neighbor cities. Baptism records, on the other hand, are carefully kept in the different churches located in the municipality, and these records include the name and sex of the children, date of birth, parent's name and locality where parents were living. The fact that only catholic churches were included may also be a limitation, but the last demographic census in Brazil in 2007 showed that 76.6% of CG population is Catholic. Baptism records may suffer from another bias, in that only live births are registered. However, this would underestimate the actual twinning prevalence in CG, and since the overall pattern of twinning is consistent between LSP and CG-LSP considering the periods of 1927–1958 and 1959–2008, it is unlikely that this would bring a significant bias to our data.

Although the importance of environmental factors cannot be formally ruled out, some factors which are important for twinning etiology such as the use of oral contraceptives, or acid folic supplementation [33]–[35] are unlikely to explain the high twinning rates already displayed by this population during the 1960s, when these substances were not available in the Brazilian market. On the other hand, high levels of IGF in milk have been implicated in elevated twinning rates [4]. Since all CG districts are based in rural economy and most of the families consume dairy products, dietary factors may represent a possible adjuvant for the high prevalence of twinning. However, because all CG districts have a similar lifestyle, milk consumption itself hardly explains the difference in twinning rates between LSP and CG-LSP. Similarly, differences in maternal age between LSP and CG-LSP are also unlikely to explain the observed pattern, since there is no statistical difference in maternal age between LSP and CG-LSP.

When one analyses extreme twinning rates in small isolates one has to keep in mind that the random fluctuations are also extreme. In Europe, a phenomenon similar to Cândido Godoi has been registered in the Aland Islands, where higher twinning rates were historically recorded compared to mainland areas of Finland and Sweden [36]. The colonization history of CG is suggestive that a genetic founder effect may have played a role in this process, leading to high shifts in allele frequency between the ancestral and the derived population. Because it is a random process, founder effects have unpredictable phenotypic effects, which, in this specific case, may be a high frequency of twinning. Our results provide strong evidence that corroborates this idea. Firstly because of the familiar aggregation of twin births and the high prevalence of DZ twins in LSP which are both indicative that genetic factors are involved in twinning in the CG population (Figure 3). Although causes for MZ twinning are still obscure, DZ twinning has a clear familial aggregation, which is taken as evidence for genetic predisposition [11]. LSP, despite being a small district with few baptisms, concentrates roughly one third of all CG twins. The increased DZ twinning proportions in LSP thus indicates that such contribution for overall twinning occurs because there are genetic factors enhancing twinning rate in CG as a whole and in LSP in particular. Secondly, strong evidence for the hypothesis of a genetic founder effect comes from the higher inbreeding coefficient found for women who gave birth to twins compared to other women.

Currently, there are several reported examples of the importance of genetic founder effects for some specific phenotypes which are more frequently in some human populations. As a general rule, a relatively high frequency of an autosomal recessive disease in an isolated population suggests a founder effect [37]. For example, it was suggested that a subgroup of Native American Athabascan populations living in Arizona and New Mexico have an unusually high incidence of severe combined immunodeficiency probably due to founder effect [38]. More recently, a case for the importance of genetic founder effects has been made for explaining the high frequency of genes responsible for ‘single-gene’ disorders and disease predispositions in Ashkenazi Jews compared to Sepharadi Jews and non-Jews [39]. Another example is the Quebec population, in Canada. This population was founded by 8500 French settlers and shows, for some genetic disease, a geographical distribution consistent with a story of serial founder effects occurred during the migration of those settlers and their descendants [40]. Is was also recently proposed that for the Sardinian population the relatively high frequency of glycogen storage disease type Ib in this population may be related to a founder effect [41].

Finally, our results also suggest that CG in general and LSP in special represent excellent population isolates where specific genetic variants influencing twin birth in humans may be identified. For the citizens of CG, our results may also be relieving, since we could formally reject the possibility that CG twins are a result of Nazi experiments and ideology. In this sense, our study illustrates how knowledge of population history and the genetic consequences may be of direct interest for the populations under study.
